# Serum glutathione peroxidase-3 concentration at diagnosis as a biomarker for assessing disease activity and damage of antineutrophil cytoplasmic antibody-associated vasculitis at diagnosis

**DOI:** 10.3389/fmolb.2025.1549454

**Published:** 2025-02-07

**Authors:** Jihye Chung, Jang Woo Ha, Yong-Beom Park, Sang-Won Lee

**Affiliations:** ^1^ Division of Rheumatology, Department of Internal Medicine, Yonsei University College of Medicine, Seoul, Republic of Korea; ^2^ Division of Rheumatology, Department of Internal Medicine, Yongin Severance Hospital, Yonsei University College of Medicine, Yongin, Gyeonggi-do, Republic of Korea; ^3^ Institute for Immunology and Immunological Diseases, Yonsei University College of Medicine, Seoul, Republic of Korea

**Keywords:** glutathione peroxidase-3, activity, inflammation, antineutrophil cytoplasmic antibody, vasculitis

## Abstract

**Background:**

In this study, we investigated whether serum glutathione peroxidase-1 (GPX-3) concentration at diagnosis could be used to assess vasculitis activity and damage at diagnosis in immunosuppressive drug-naïve patients with antineutrophil cytoplasmic antibody (ANCA)-associated vasculitis (AAV).

**Methods:**

We included 71 immunosuppressive drug-naïve patients newly diagnosed with AAV. Medical records were retrospectively reviewed and serum GPX-3 concentration was measured using serum samples collected and stored at diagnosis. The degree of vascular activity and extent of damage were assessed using the Birmingham vasculitis activity score (BVAS) and vasculitis damage index (VDI), respectively. Poor outcomes including all-cause mortality, end-stage kidney disease, and cerebrovascular and cardiovascular diseases were also investigated.

**Results:**

The median age of the study subjects was 63.0 years, 26 and 45 patients were males and females, respectively. The median GPX-3 concentration was measured as 82.8 ng/mL. Serum GPX-3 concentration at diagnosis was inversely correlated with BVAS (r = −0.280), VDI (r = −0.263), and C-reactive protein (r = −0.261) at diagnosis, whereas, it was positively correlated with haemoglobin (r = 0.255), and serum albumin (r = 0.240) at diagnosis, respectively. However, serum GPX-3 concentration at diagnosis was not significantly associated with poor outcomes during follow-up in patients with AAV.

**Conclusion:**

In this study, we demonstrated for the first time that serum GPX-3 concentration at diagnosis correlates with vasculitis activity and damage at diagnosis in patients with AAV, suggesting a possible role of serum GPX-3 as a complementary biomarker for assessing AAV activity in real clinical practice.

## 1 Introduction

Glutathione peroxidases (GPXs) are a group of enzymes that can detoxify hydrogen peroxide to produce other free radicals (Handy and Loscalzo, 2022). Glutathione peroxidase-3 (GPX-3) is one of the 8 isozymes of GPX and particularly, as plasma GPX because it is mainly distributed extracellularly unlike other GPX isozymes (Rush and Sandiford, 2003). Several studies were measuring serum GPX-3 concentrations and investigating the clinical significance in patients with various inflammatory or cancerous diseases. Low serum GPX-3 concentration is considered a biomarker to assess a high degree of inflammation or poor prognosis (Bierl et al., 2004; Manzanares et al., 2009; Agnani et al., 2011), which might be attributed to be due to the clinical properties of GPX-3 as an antioxidant enzyme (Chang et al., 2020). Antineutrophil cytoplasmic antibody (ANCA)-associated vasculitis (AAV) is a systemic vasculitis that invades the smallest units of arteries, veins, and capillaries and may potentially affect almost all major organs (Jennette et al., 2013; Watts et al., 2007). According to the clinical, laboratory, radiological, and histological features, AAV is divided into three subtypes: microscopic polyangiitis (MPA), granulomatosis with polyangiitis (GPA), and eosinophilic GPA (EGPA) (Suppiah et al., 2022; Robson et al., 2022; Grayson et al., 2022). Although three subtypes are essentially characterized by each subtype’s predominant features, given the affected vessel system, they may share the common clinical manifestations related to the three typical major organs with the largest surface area of capillaries such as the kidneys, lungs, and skin (Kronbichler et al., 2024). On the other hand, the pathogenesis of AAV involves many immunological processes and responses, and particularly, the role of reactive oxygen species (ROS) is known to contribute significantly to vascular inflammation and damage (Kitching et al., 2020; Choi et al., 2019). Therefore, it can be reasonably assumed that if the expression of GPX isozymes with a peroxidase function to remove ROS is reduced, the degree of vascular inflammation and damage may increase. Moreover, it could be speculated that serum GPX-3 concentration may be inversely associated with the current degree of vascular inflammation and damage in patients with AAV. However, no study has elucidated the clinical role of serum GPX-3 concentration in assessing the current degree of vascular inflammation and damage in patients with AAV. Hence, in this study, we investigated whether serum GPX-3 concentration at diagnosis could be used to assess vasculitis activity and damage at diagnosis in immunosuppressive drug-naïve patients with AAV.

## 2 Materials and methods

### 2.1 Patients

We randomly selected 71 immunosuppressive drug-naïve patients newly diagnosed with AAV from an observational single-centre cohort of Korean patients with AAV at the University Tertiary Hospital and included them in this study. The inclusion criteria were i) fulfilment of the 2007 European Medicine Agency algorithm for AAV, the 2022 revised Chapel Hill Consensus Conference nomenclature of vasculitides, and the 2022 American College of Rheumatology and European Alliance of Associations for Rheumatology classification criteria for MPA, GPA, and EGPA (Handy and Loscalzo, 2022; Rush and Sandiford, 2003; Suppiah et al., 2022; Robson et al., 2022; Grayson et al., 2022); ii) first diagnosis of AAV by the specialised Rheumatologists in this tertiary hospital; iii) sufficiently documented medical records for collecting clinical, laboratory, radiological, and histological data; iv) well-prepared sera obtained on patients’ consent and stored at diagnosis; v) follow-up duration for 6 months or greater after diagnosis; vii) no serious concomitant medical conditions mimicking AAV such as malignancies, severe infectious diseases requiring hospitalization, and autoantibody-medicated autoimmune diseases, or inducing ANCA false positive at diagnosis; vii) no exposure to immunosuppressive drugs for AAV treatment at least within 4 weeks before diagnosis. This study was approved by the Institutional Review Board (IRB) of Severance Hospital, Seoul, Republic of Korea (IRB number 4-2016-0901) and, when required, written informed consent was obtained from patients at the time of blood sampling. The IRB waived the need for written informed consent when it was previously obtained at entry into the SHAVE cohort.

### 2.2 Blood sampling and consent form

On the day of AAV diagnosis, informed consent was obtained, AAV-specific indices were assessed, and whole blood was collected from patients with AAV. Sera were immediately isolated from whole blood and stored at −80°C on the day of AAV diagnosis.

### 2.3 Clinical data at diagnosis

Age, sex, smoking history, and body mass index (BMI) were recorded. Data on AAV-subtype, ANCA type and positivity, and AAV-specific indices were also collected. In the present study, myeloperoxidase (MPO)-ANCA and proteinase 3 (PR3)-ANCA measured using an immunoassay as well as perinuclear (P)-ANCA and cytoplasmic (C)-ANCA detected using an indirect immunofluorescence assay with ethanol fixation were all recognised as ANCA test results according to the 2022 criteria (Suppiah et al., 2022; Robson et al., 2022; Grayson et al., 2022). AAV-specific indices included the Birmingham vasculitis activity score (BVAS), the five-factor score (FFS), the 36-Item Short Form Survey (SF-36) physical and mental component summaries (PCS and MCS), and the vasculitis damage index (VDI) (Mukhtyar et al., 2009; Guillevin et al., 2011; Flossmann et al., 2007; Han et al., 2004). In this study, the degrees of vascular activity and the extent of damage were expressed using BVAS and VDI, respectively. Routinely performed laboratory test results, including those of acute-phase reactants such as erythrocyte sedimentation rate (ESR) and C-reactive protein (CRP) tests, were recorded.

### 2.4 Measurement of serum GPX-3 concentration at diagnosis

Serum GPX-3 concentration was assessed from stored sera at diagnosis using enzyme-linked immunosorbent assay kits (Mybiosource, San Diego, CA, United States) according to the manufacturer’s instructions. In this study, a continuous variable of serum GPX-3 concentration was used for statistical analyses.

### 2.5 Clinical data during follow-up

All-cause mortality, end-stage kidney disease (ESKD), cerebrovascular accident (CVA), and acute coronary syndrome (ACS) were considered poor AAV outcomes during follow-up. The follow-up duration based on each poor outcome was defined as the period from diagnosis to its occurrence in patients with a corresponding poor outcome, whereas the duration from diagnosis to the last visit was defined for those without. Medications administered after AAV diagnosis and during the disease course, including glucocorticoids and immunosuppressive drugs, were also assessed. Accordingly, ESKD, CVA, and ACS that occurred before diagnosis were not considered poor AAV outcomes in the present study.

### 2.6 Statistical analyses

All statistical analyses were performed using the SPSS version 26 (IBM Corporation, Armonk, NY, United States) for Windows (Microsoft Corporation, Redmond, WA, United States). Continuous and categorical variables are expressed as medians (interquartile range, Q1−Q3), and numbers (percentages). The Mann-Whitney U test was used to compare significant differences between continuous variables. The correlation coefficients (r) between the two variables were determined using Pearson’s correlation analysis. Cox proportional hazards model analysis was performed to obtain the hazard ratio (HR) of serum GPX-3 concentration at diagnosis for each poor outcome during follow-up. A significant area under the curve (AUC) was determined using receiver operating characteristic (ROC) curve analysis. A comparison of the cumulative survival rates between the two groups was performed using Kaplan Meier survival analysis with the log-rank test. Statistical significance was set at P < 0.05.

## 3 Results

### 3.1 Characteristics of patients with AAV at diagnosis

The median age of the study subjects was 63.0 (51.0–74.0) years, and 26 and 45 patients were males and females, respectively. Two patients were ex-smokers and the median BMI was 22.4 (21.1–24.8) kg/m^2^. Of these 71 patients, 35, 23, and 13 were diagnosed with MPA, GPA, and EGPA, respectively. The median BVAS, FFS, SF-36 PCS and MCS, and VDI values were 5.0 (3.0–17.0), 0 (0–1.0), 52.5 (34.4–68.1), 58.4 (40.0–73.4), and 3.0 (2.0–4.0), respectively. The median ESR and CRP were 21.0 (7.0–85.3) mm/h and 3.8 (0.9–29.1) mg/L, respectively. The median GPX-3 concentration was measured as 82.8 (44.3–156.0) ng/mL (Table 1).

**TABLE 1:** Characteristics of patients with AAV at diagnosis (N = 71).

Variables	Values
Demographic data	
Age (years)	63.0 (51.0–74.0)
Male sex [N, (%)]	26 (36.6)
Female sex [N, (%)]	45 (63.4)
Ex-smoker [N, (%)]	2 (2.8)
Body mass index (kg/m^2^)	22.4 (21.1–24.8)
AAV subtype [N, (%)]	
MPA	35 (49.3)
GPA	23 (32.4)
EGPA	13 (18.3)
ANCA type and positivity [N, (%)]	
MPO-ANCA (or P-ANCA) positive	41 (57.7)
PR3-ANCA (or C-ANCA) positive	11 (15.5)
Both ANCA positive	3 (4.2)
AAV-specific indices	
BVAS	5.0 (3.0–17.0)
FFS	0 (0–1.0)
SF-36 PCS	52.5 (34.4–68.1)
SF-36 MCS	58.4 (40.0–73.4)
VDI	3.0 (2.0–4.0)
Comorbidities [N, (%)]	
Type 2 diabetes mellitus	15 (21.1)
Hypertension	23 (32.4)
Dyslipidaemia	13 (18.3)
Routinely performed laboratory test results	
ESR (mm/hr)	21.0 (7.0–85.3)
CRP (mg/L)	3.8 (0.9–29.1)
White blood cell count (/mm^3^)	7,500.0 (5,930.0–9,640.0)
Haemoglobin (g/dL)	12.4 (10.4–13.6)
Platelet count (x 1,000/mm^3^)	243.5 (193.0–354.8)
Fasting glucose (mg/dL)	94.0 (88.0–109.0)
Blood urea nitrogen (mg/dL)	19.2 (13.1–28.7)
Serum creatinine (mg/dL)	0.8 (0.6–1.5)
Total serum protein (g/dL)	6.8 (6.4–7.4)
Serum albumin (g/dL)	4.2 (3.7–4.4)
Alkaline phosphatase (IU/L)	70.0 (58.0–92.0)
Aspartate transaminase (IU/L)	20.5 (16.0–25.8)
Alanine transaminase (IU/L)	16.5 (11.0–26.0)
Serum GPX-3 (ng/mL)	82.8 (44.3–156.0)

Values are expressed as a median (25–75 percentile) or N (%).

ANCA, antineutrophil cytoplasmic antibody; AAV, ANCA-associated vasculitis; MPA, microscopic polyangiitis; GPA, granulomatosis with polyangiitis; EGPA, eosinophilic GPA; MPO, myeloperoxidase; P, perinuclear; PR3, proteinase 3; C, cytoplasmic; BVAS, the Birmingham vasculitis activity score; FFS, the five-factor score; SF-36, the 36-Item Short Form Survey; PCS, physical component summary; MCS, mental component summary; VDI, the vasculitis damage index; ESR, erythrocyte sedimentation rate; CRP, C-reactive protein; GPX-3, glutathione peroxidase-3.

### 3.2 Correlation analysis of serum GPX-3 concentration and continuous variables at diagnosis in patients with AAV

Serum GPX-3 concentration at diagnosis was inversely correlated with BVAS (r = −0.280, P = 0.018), VDI (r = −0.263, P = 0.029), and CRP (r = −0.261, P = 0.028) at diagnosis, whereas, it was positively correlated with haemoglobin (r = 0.255, P = 0.032), and serum albumin (r = 0.240, P = 0.045) at diagnosis, respectively. Additionally, both SF-36 PCS and ESR tended to correlate with serum GPX-3 concentration simultaneously but the correlation was not statistically significant (Table 2).

**TABLE 2:** Correlation analysis of serum GPX-3 concentration and continuous variables at diagnosis in patients with AAV.

Variables	Correlation coefficient (r)	*P-value*
Demographic data		
Age (years)	−0.054	0.652
Body mass index (kg/m^2^)	0.013	0.917
AAV-specific indices		
BVAS	−0.280	0.018
FFS	−0.131	0.277
SF-36 PCS	0.219	0.067
SF-36 MCS	0.197	0.100
VDI	−0.263	0.029
Acute-phase reactants		
ESR (mm/hr)	−0.217	0.071
CRP (mg/L)	−0.261	0.028
Routinely performed laboratory test results		
White blood cell count (/mm^3^)	−0.107	0.374
Haemoglobin (g/dL)	0.255	0.032
Platelet count (x 1,000/mm^3^)	−0.120	0.322
Fasting glucose (mg/dL)	−0.132	0.303
Blood urea nitrogen (mg/dL)	0.021	0.862
Serum creatinine (mg/dL)	−0.051	0.675
Total serum protein (g/dL)	0.168	0.166
Serum albumin (g/dL)	0.240	0.045
Alkaline phosphatase (IU/L)	−0.048	0.691
Aspartate transaminase (IU/L)	0.047	0.703
Alanine transaminase (IU/L)	0.129	0.288

GPX-3, glutathione peroxidase-3; ANCA: antineutrophil cytoplasmic antibody; AAV, ANCA-associated vasculitis; BVAS: the Birmingham vasculitis activity score; FFS, the five-factor score; SF-36, the 36-Item Short Form Survey; PCS, physical component summary; MCS, mental component summary; VDI, the vasculitis damage index; ESR, erythrocyte sedimentation rate; CRP, C-reactive protein.

### 3.3 Comparison of serum GPX-3 concentration between the two groups

**FIGURE 1:**
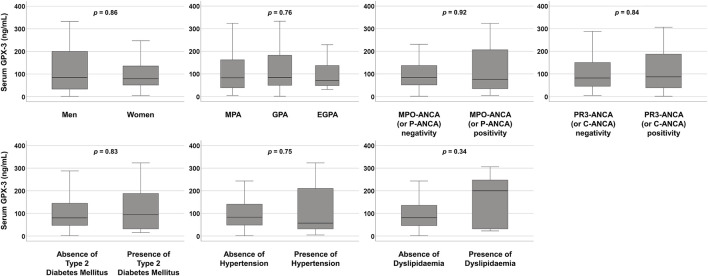
Comparison of serum GPX-3 concentrations. No significant differences between the two groups were observed. GPX-3, glutathione peroxidase-3; MPA, microscopic polyangiitis; GPA, granulomatosis with polyangiitis; EGPA, eosinophilic GPA; MPO, myeloperoxidase; ANCA, antineutrophil cytoplasmic antibody; P, perinuclear; PR3, proteinase 3: C, cytoplasmic.

At the time of diagnosis, serum GPX-3 concentrations according to sex, AAV subtype, MPO-ANCA (or P-ANCA) positivity, PR3-ANCA (or C-ANCA) positivity, and comorbidities were compared but no significant differences between the two groups were observed (Figure 1). Additionally, since serum GPX-3 concentration was inversely correlated with BVAS, we divided the study participants into two groups according to each of the nine systemic items of BVAS and compared serum GPX-3 concentrations between the two groups (Mukhtyar et al., 2009). We found that only serum GPX-3 concentrations, according to general manifestation, were significantly different between the two groups (55.8 ng/mL for patients with general manifestation vs. 84.8 ng/mL for those without, P = 0.021) (Table 3).

**TABLE 3:** Comparative analysis of serum GPX-3 according to the presence of each systemic item of BVAS in patients with AAV.

Systemic item of BVAS	Median serum GPX-3		*P-value*
	Absence	Presence	
General manifestation	84.8 ng/mL	55.8 ng/mL	0.021
Cutaneous manifestation	83.9 ng/mL	63.2 ng/mL	0.247
Mucous and ocular manifestation	81.3 ng/mL	83.4 ng/mL	0.694
Otorhinolaryngologic manifestation	88.6 ng/mL	68.9 ng/mL	0.054
Pulmonary manifestation	86.5 ng/mL	71.3 ng/mL	0.070
Cardiovascular manifestation	83.9 ng/mL	57.6 ng/mL	0.283
Gastrointestinal manifestation	N/A	N/A	N/A
Renal manifestation	80.1 ng/mL	84.3 ng/mL	0.972
Nervous systemic manifestation	84.3 ng/mL	82.0 ng/mL	0.568

GPX-3, glutathione peroxidase-3; BVAS, the Birmingham vasculitis activity score; ANCA, antineutrophil cytoplasmic antibody; AAV, ANCA-associated vasculitis; N/A, not applicable.

### 3.4 Poor outcomes and immunosuppressive drugs administered during follow-up in patients with AAV

During follow-up after diagnosis, among the 71 patients, 6 (8.5%) died, and 17, four, and one had ever experienced and suffered from ESKD, CVA, and ACS, respectively. Glucocorticoids were administered to 70 (98.6%) patients. Among the intravenous induction therapeutic regimens, cyclophosphamide, and rituximab were administered to 47 (66.2%), and 13 (18.3%) patients, respectively. Among oral immunosuppressive drugs, the most frequently administered was azathioprine (57.7%), followed by mycophenolate mofetil (26.8%) (Table 4).

**TABLE 4:** Poor outcomes and immunosuppressive drugs administered during follow-up in patients with AAV (N = 71).

Variables	Values
Poor outcomes [N, (%)]	
All-cause mortality	6 (8.5)
ESKD	17 (23.9)
CVA	4 (5.6)
ACS	1 (1.4)
Follow-up periods based on each poor outcomes (months)	
All-cause mortality	26.1 (10.8–45.7)
ESKD	24.3 (8.3–45.7)
CVA	25.8 (8.5–42.2)
ACS	25.8 (9.1–42.2)
Immunosuppressive drugs administered [N, (%)]	
Glucocorticoids	70 (98.6)
Cyclophosphamide	47 (66.2)
Rituximab	13 (18.3)
Mycophenolate mofetil	19 (26.8)
Azathioprine	41 (57.7)
Tacrolimus	6 (8.5)
Methotrexate	3 (4.2)

Values are expressed as a median (25–75 percentile) or N (%).

ANCA, antineutrophil cytoplasmic antibody; AAV, ANCA-associated vasculitis; ESKD, end-stage kidney disease; CVA, cerebrovascular accident; ACS, acute coronary syndrome.

### 3.5 Cox analysis of serum GPX-3 concentration for each poor outcome

Serum GPX-3 concentration at diagnosis was not significantly associated with all-cause mortality, ESKD, CVA, or ACS during follow-up in patients with AAV (Table 5).

**TABLE 5:** Univariable Cox proportional hazards analysis of serum GPX-3 concentration at diagnosis for each poor outcome during follow-up in patients with AAV.

Poor outcomes	HR	95% CI	*P-value*
All-cause mortality	0.998	0.988–1.008	0.694
ESKD	0.999	0.993–1.005	0.751
CVA	0.997	0.984–1.010	0.640
ACS	1.014	0.992–1.038	0.218

GPX-3, glutathione peroxidase-3; ANCA, antineutrophil cytoplasmic antibody; AAV, ANCA-associated vasculitis; ESKD, end-stage kidney disease; CVA, cerebrovascular accident; ACS, acute coronary syndrome.

## 4 Discussion

Considering the clinical utility of low serum GPX-3 concentration (or GPX-3 deficiency) in various diseases, in this study we investigated whether serum GPX-3 concentration at diagnosis could be used to assess vasculitis activity and damage at diagnosis in immunosuppressive drug-naive patients with AAV, and obtained some interesting findings. First, significant correlations were observed between serum GPX-3 concentration at diagnosis and both BVAS and VDI at diagnosis, indicating that serum GPX-3 can be a potential biomarker to assess vasculitis activity and damage at diagnosis in patients newly diagnosed with AAV. Additionally, serum GPX-3 had another potential to reflect the inflammatory burden as much as traditional acute-phase reactants such as CRP and serum albumin. However, serum GPX-3 did not predict poor AAV outcomes during follow-up. Therefore, in the present study, we demonstrated that serum GPX-3 concentration at diagnosis has the potential to be a novel biomarker that can assess and estimate activity and damage caused by AAV, although it is confined to the time of diagnosis.

ROS are well-known mediators of the pathophysiological signal transduction of inflammation processes (Forrester et al., 2018). At the site of inflammation, the increased ROS production by polymorphonuclear cells may induce endothelial cell dysfunction and tissue damage. Furthermore, the combination of endothelial impairment and oxidative stress may loosen the endothelial junction of the blood-tissue barrier and accelerate the extravascular migration of inflammatory immune cells to the inflamed tissue (Mittal et al., 2014). Similarly, in the pathophysiology of AAV, ROS is also known to play an important role in the development and exacerbation of AAV (Kitching et al., 2020; Choi et al., 2019). GPX-3 can reduce oxidative stress by scavenging ROS, which may reduce the nuclear translocation of transcription factors such as the p65 unit of nuclear factor kappa-light-chain-enhancer of activated B cells, ultimately suppressing the expression of inflammatory mediators located downstream of the promoter sites (An et al., 2018; Nirgude and Choudhary, 2021). Based on these findings, it is reasonable to infer that decreased GPX-3 expression and production may increase vascular activity and damage owing to a diminished effect in suppressing the transcription of inflammatory mediators. This inference supports the key finding of this study that serum GPX-3 concentration at diagnosis was inversely correlated with BVAS, VDI, and CRP at diagnosis.

We investigated which item among the nine systemic items of BVAS based on major organ involvement contributed to the inverse correlation with serum GPX-3 concentration. We divided the study participants into two groups according to each of the nine systemic items of BVAS and compared serum GPX-3 concentrations between the two groups (Mukhtyar et al., 2009). Only serum GPX-3 concentrations, according to general manifestation, was significantly different between the two groups. Additionally, otorhinolaryngologic (68.9 ng/mL vs. 88.6 ng/mL, P = 0.054) and pulmonary (71.3 ng/mL vs. 86.5 ng/mL, P = 0.070) manifestations showed significant differences between the two groups but they did not reach statistical significance. Although GPX-3 has been reported to be associated with renal pathological findings (Burk et al., 2011), this study was not able to reveal an association between serum GPX-3 concentration and kidney involvement of AAV (Table 3). As per the general manifestation, since its minor items were closer to constitutional symptoms, such as myalgia, arthralgia/arthritis, fever, and weight loss, rather than those limited to a specific organ (Mukhtyar et al., 2009), this subgroup analysis was not sufficient to explain the inverse correlation between serum GPX-3 concentration and BVAS. Therefore, we conclude that the correlation between serum GPX-3 concentration and BVAS may be based on the sum of the cumulative contributions of several organ involvements, such as the ear, nose, throat or lungs, rather than that of specific organ invasion by AAV.

In the present study, serum GPX-3 concentration at diagnosis could not predict poor outcomes during follow-up in patients with AAV. Given that the risk factors for all-cause mortality among the four poor outcomes were relatively well established, to verify the methodological error of the Cox analysis for the results in Table 4 results, we performed univariable and multivariable Cox proportional hazards analyses including traditional, AAV-specific, and inflammation-related risk factors along with GPX-3 concentration at diagnosis for all-cause mortality during follow-up (Murray et al., 2013; Tan et al., 2017; Mukhtyar et al., 2008). In univariable Cox analysis, dyslipidaemia, VDI, white blood cell count, haemoglobin, and serum albumin were significantly associated with all-cause mortality in patients with AAV; however, serum GPX-3 concentration was not. Nevertheless, to investigate the predictive and independent potential of serum GPX-3 concentration for all-cause mortality, we included serum GPX-3 concentration in multivariable Cox analysis. However, in multivariable Cox analysis, only dyslipidaemia was independently associated with all-cause mortality (Supplementary Table S1). Therefore, we verified that there was no methodological error in the Cox analysis, and demonstrated that the independent predictive potential of serum GPX-3 concentration for all-cause mortality was not notable.

Additionally, although a continuous value of serum GPX-3 concentration did not show significant predictive potential for poor outcomes, we wondered whether a categorical value of serum GPX-3 concentration according to its cut-offs of poor outcomes such as all-cause mortality, ESKD, CVA, and ACS could be a significant predictor of those poor outcomes. We attempted to obtain each optimal cut-off of serum GPX-3 concentration for each poor outcome using the receiver operating characteristic ROC curve analyses; however, we found no significant areas under the curve (AUC) of serum GPX-3 concentration for poor outcomes. Therefore, we failed to obtain the optimal cut-offs of serum GPX-3 concentration for all-cause mortality, ESKD, CVA, or ACS in this study (Supplementary Figure S1). Meanwhile, among the four poor outcomes, despite no statistical significance, all-cause mortality showed a relatively higher AUC compared to the remaining ESKD, CVA, and ACS. When the patients were divided into two groups such as the higher and lower groups according to the median value of GPX-3 concentration of 82.8 ng/mL, in Kaplan Meier survival analysis, however, no significant difference in cumulative patients’ survival rates between patients in the higher and lower groups was observed (Supplementary Figure S2). We concluded that serum GPX-3 can be useful in estimating the current activity and damage of vasculitis but not anticipating future poor outcomes in patients with AAV. This study has the advantage that this is the first to demonstrate the significant correlations between serum GPX-3 concentration with activity (BVAS) and damage (VDI) at diagnosis in patients with AAV.

Antiphospholipid syndrome (APS) is a systemic autoimmune disease characterized by thrombotic, non-thrombotic, and obstetric manifestations in individuals with persistent antiphospholipid antibodies (aPL). These antibodies are commonly detected through immunoassays for anticardiolipin (aCL) and anti-β2-glycoprotein-I (aβ2GPI) antibodies, or lupus anticoagulant tests. The interaction between aPL and targets like β2GPI triggers the activation of endothelial cells, platelets, monocytes, and neutrophils, as well as the complement and coagulation systems, forming a key molecular mechanism underlying APS pathogenesis (Knight and Erkan, 2024). This interaction occurs at specific microdomains of the cell plasma membrane known as lipid rafts, which are rich in glycosphingolipids and cholesterol (Capozzi et al., 2023). Recent reviews highlight that Mitogen-Activated Protein Kinases (MAPKs) play a crucial role in transmitting signals from the cell surface to the nucleus, regulating gene activity. Among the four main branches of the MAPK pathway, p38MAPK is a key regulator in APS. Additionally, aPL stimulates the production of ROS in endothelial cells. These ROS act as secondary messengers, activating the p38MAPK pathway, which is involved in the pathogenesis of APS (Feng et al., 2024). GPX-3 is an antioxidant enzyme that plays a critical role in removing ROS, and thus, APS patients may exhibit a decrease in serum GPX-3 concentration. However, this study did not include patients with aPL antibodies, and therefore, subgroup analysis related to this aspect could not be performed.

## 5 Limitations

This study has several limitations. The most critical limitation was that the number of participating patients was not sufficiently large to generalize these results and apply them to real clinical practice and that clinical data and sera samples from the time of diagnosis were used retrospectively. Additionally, although this study focused on the clinical implications of serum GPX-3 concentration among patients with AAV only, this study could not provide the results of the comparative analysis of serum GPX-3 concentration between AAV patients and age- and sex-matched healthy controls. However, given that this is the first pilot study to explore the clinical utility of serum GPX-3 concentration in patients with AAV, we believe that this study has clinical implications in that this identified a novel biomarker that can assess and estimate activity and damage caused by AAV at diagnosis, although confined to the time of diagnosis. A prospective future study enrolling more patients and equipping serial clinical data and paired serum samples will provide more reliable and dynamic information on the clinical significance of serum GPX-3 concentration for not only assessing activity and damage at diagnosis but also predicting and monitoring the prognosis during follow-up in patients with AAV.

## 6 Conclusion

In this study, we demonstrated for the first time that serum GPX-3 concentration at diagnosis correlates with vasculitis activity and damage at diagnosis in patients with AAV, suggesting a possible role of serum GPX-3 as a complementary biomarker for assessing AAV activity in real clinical practice.

## Data Availability

The raw data supporting the conclusions of this article will be made available by the authors, without undue reservation.
