# Supplementary material for: Serum glutathione peroxidase-3 concentration at diagnosis as a biomarker for assessing disease activity and damage of antineutrophil cytoplasmic antibody-associated vasculitis at diagnosis
Source: Front Mol Biosci. 2025 Feb 7;12:1549454. doi: 10.3389/fmolb.2025.1549454 (PMC11842223; doi:10.3389/fmolb.2025.1549454)
Supplement: Supplementary file 1 [file Table1.docx]

**Supplementary Table 1. Cox proportional hazards analysis of traditional, AAV-specific, and inflammation-related risk factors at diagnosis for all-cause mortality during follow-up in patients with AAV**

| **Variables** | ***Univariable*** | | | ***Multivariable*** | | |
| --- | --- | --- | --- | --- | --- | --- |
|  | **HR** | **95% CI** | ***P-value*** | **HR** | **95% CI** | ***P-value*** |
| **Traditional risk factors** |  |  |  |  |  |  |
| Age | 1.100 | 0.998, 1.212 | 0.054 |  |  |  |
| Male sex | 3.500 | 0.641, 19.108 | 0.148 |  |  |  |
| Body mass index | 1.178 | 0.917, 1.512 | 0.199 |  |  |  |
| Type 2 diabetes mellitus | 4.156 | 0.838, 20.607 | 0.081 |  |  |  |
| Hypertension | 1.156 | 0.211, 6.314 | 0.867 |  |  |  |
| Dyslipidaemia | 10.620 | 1.941, 58.121 | 0.006 | 39.138 | 2.383, 642.759 | 0.010 |
| **AAV-specific risk factors** |  |  |  |  |  |  |
| BVAS | 1.071 | 0.994, 1.155 | 0.073 |  |  |  |
| FFS | 2.335 | 0.896, 6.086 | 0.083 |  |  |  |
| VDI | 1.574 | 1.066, 2.325 | 0.023 | 0.921 | 0.447, 1.899 | 0.824 |
| **Inflammation-related risk factors** |  |  |  |  |  |  |
| ESR | 1.014 | 0.997, 1.032 | 0.110 |  |  |  |
| CRP | 1.014 | 0.998, 1.031 | 0.083 |  |  |  |
| White blood cell count | 1.203 | 1.065, 1.359 | 0.003 | 1.247 | 0.913, 1.702 | 0.165 |
| Haemoglobin | 0.571 | 0.362, 0.899 | 0.016 | 0.859 | 0.405, 1.820 | 0.691 |
| Platelet count | 1.003 | 0.998, 1.008 | 0.253 |  |  |  |
| Blood urea nitrogen | 1.050 | 0.992, 1.112 | 0.091 |  |  |  |
| Serum creatinine | 1.481 | 0.918, 2.387 | 0.107 |  |  |  |
| Serum total protein | 0.405 | 0.161, 1.016 | 0.054 |  |  |  |
| Serum albumin | 0.155 | 0.050, 0.484 | 0.001 | 0.316 | 0.034, 2.973 | 0.314 |
| Serum GPX-3 concentration | 0.998 | 0.998, 1.008 | 0.694 | 0.992 | 0.979, 1.006 | 0.273 |

ANCA: antineutrophil cytoplasmic antibody; AAV: ANCA-associated vasculitis; BVAS: the Birmingham vasculitis activity score; FFS: the five-factor score; VDI: the vasculitis damage index; ESR: erythrocyte sedimentation rate; CRP: C-reactive protein; GPX-3: glutathione peroxidase-3.
