# Supplementary figures and images for: Serum glutathione peroxidase-3 concentration at diagnosis as a biomarker for assessing disease activity and damage of antineutrophil cytoplasmic antibody-associated vasculitis at diagnosis
Source: Front Mol Biosci. 2025 Feb 7;12:1549454. doi: 10.3389/fmolb.2025.1549454 (PMC11842223; doi:10.3389/fmolb.2025.1549454)

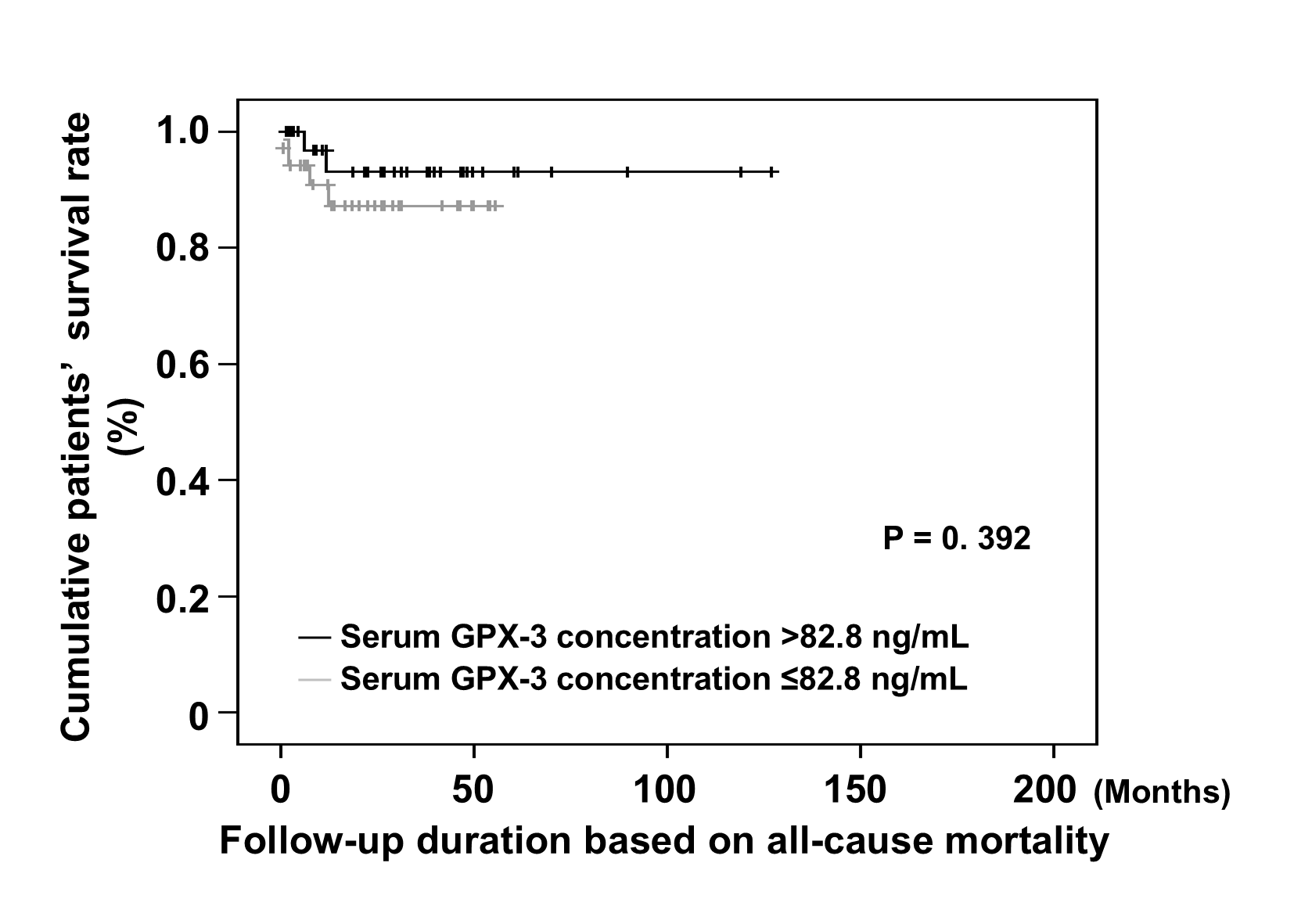

Supplement: Supplementary file 2 [file Image2.TIF]

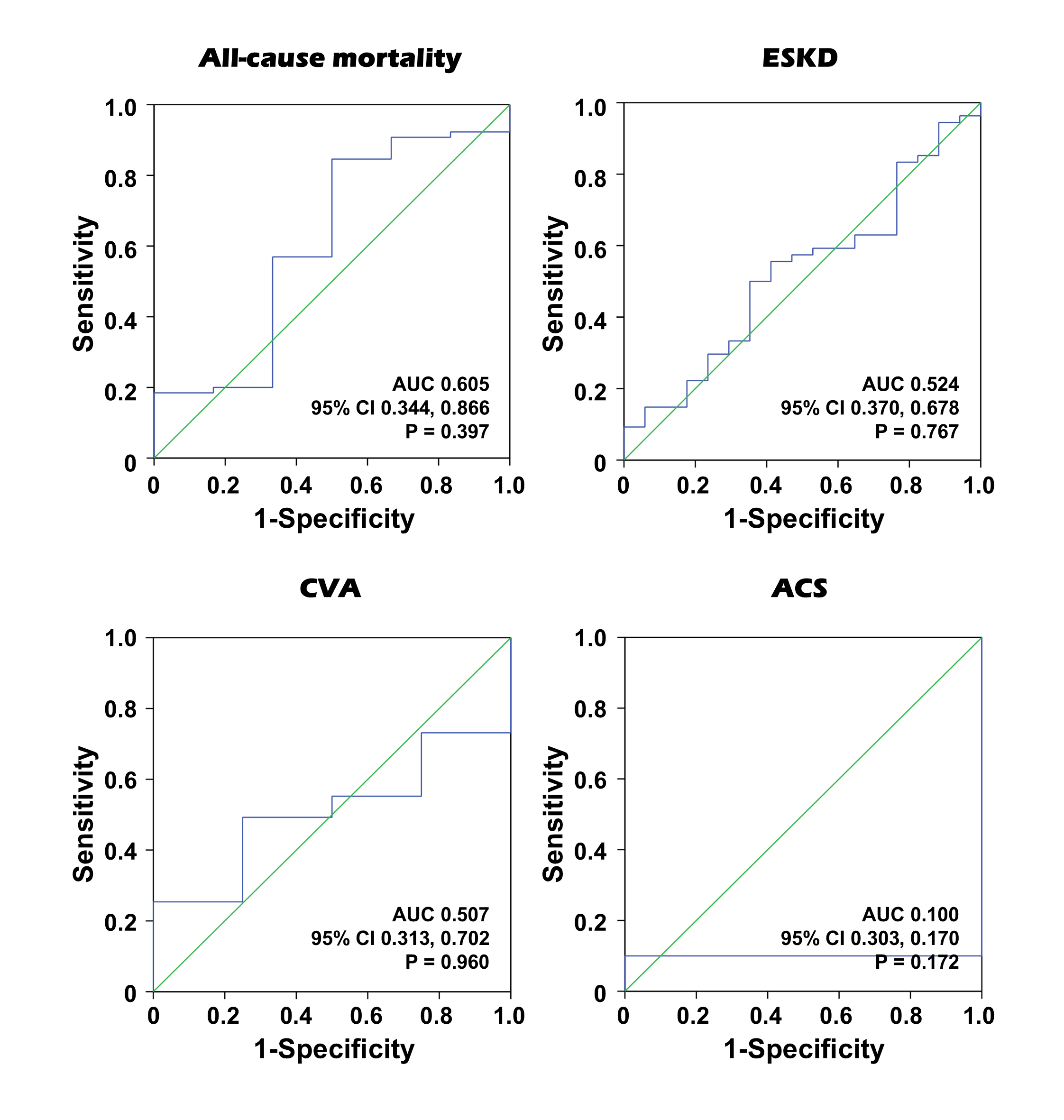

Supplement: Supplementary file 3 [file Image1.TIF]
